# Translation and Cultural Adaptation of the Creighton Model FertilityCare™ System Follow-up Form to Brazilian Portuguese

**DOI:** 10.61622/rbgo/2025rbgo48

**Published:** 2025-07-15

**Authors:** Carolina de Souza Delage Faria, Carla Ferreira Kikuchi Fernandes, José Maria Cordeiro Ruano, Marair Gracio Ferreira Sartori

**Affiliations:** 1 Escola Paulista de Medicina da Universidade Federal de São Paulo Department of Gynecology and Obstetrics SP Brazil Department of Gynecology and Obstetrics, Escola Paulista de Medicina da Universidade Federal de São Paulo, SP, Brazil.

**Keywords:** Creighton Model System, Family planning, Cross-cultural comparison, Surveys and questionnaires

## Abstract

**Objective:**

To develop the Brazilian Portuguese version of the Follow-up Form for the Creighton Model FertilityCare™ System.

**Methods:**

Translation and cultural adaptation of the Follow-up Form for use in Brazil, in 6 steps: Translation, Expert Panel, Back-Translation, Pre-test, Review, and Final Version, according to the World Health Organization methodology.

**Results:**

The 25 sections comprising the Follow-up Form were translated with 14 sections undergoing an adaptation process in one of the stages. In order to maximize semantic, idiomatic, experiential, and conceptual equivalence of the items from the original English version to Portuguese. The need for adaptation was due to four reasons: first, the format of paired and seemingly repetitive questions. Second, the difference in cultural reality, such as hygiene and consumption habits, between the United States and Brazil. Third, the use of technical terms, medical vocabulary. And fourth, sentences that contain many concepts related to the use of the Creighton Model FertilityCare™ System. The sample included 127 Creighton Model FertilityCare™ System users, with an average age of 33.7 years, and 88.2% were married. The majority, 68 (53.5%), were not using any family planning method when they started Creighton Model FertilityCare™ System 49.2% were trying to conceive in the past year.

**Conclusion:**

The translation of the Follow-up Form into Brazilian Portuguese resulted in a final version that maintained the intercultural and conceptual equivalence to the original English version. This instrument can be used by all practitioners in Brazil with the assurance that the standardization in the application of Creighton Model FertilityCare™ System reflects the original purpose of the method.

## Introduction

The Creighton Model FertilityCare™ System (CrMS) is a Natural Fertility Appreciation Method developed in 1976 by gynecologist and obstetrician Dr. Thomas Hilgers, based on research conducted at St. Louis University and Creighton University.^(1)^ This method is based on the observation and recording of vaginal discharge to identify the periods of fertility and infertility in a woman's menstrual cycle. The pattern of the discharge is recorded on a specific CrMS chart, allowing the woman to monitor her fertility and gynecological health.^(2)^

The method, when applied correctly, provides important information about the woman's health, with many details.^(3)^ For example, whether the vaginal discharge is in a physiological or imbalanced pattern; whether ovulation is actually occurring; whether the hormonal production of the fertile cycle is adequate;^(4)^ whether there is any type of abnormal bleeding during or outside the menstrual period; among others. Therefore, it allows the woman to track and monitor her fertility and gynecological health.^(5–7)^

By understanding the method, the couple becomes capable of deciding and choosing whether to use CrMS with the aim of achieving or delaying (avoiding) pregnancy.^(8)^ If learned and applied correctly, it allows the woman to identify her ovulation with 95.4% accuracy within a range of approximately two days. It is a reliable method both for delaying and achieving pregnancy.^(9–11)^

The CrMS is based on the delivery of a standardized educational content to its educators, practitioners, physicians, and users, known as Natural Procreation Education, and it is the only cycle charting model with specific medical applications.^(12,13)^ Standardization is possible thanks to the coordinated and integrated educational tools in the teaching system, some of which are:

1. The Picture Dictionary of the CREIGHTON MODEL FertilityCare™ System - presents the observation of mucus.^(14)^
2. The CREIGHTON MODEL System chart - user's worksheet.^(2)^
3. The Vaginal Discharge Recording System (VDRS) - standardizes the observation of vaginal discharge and allows the use of standardized terminology and recording system.^(14)^
4. The CREIGHTON MODEL System stamps - stickers used to make cycle information more visual.^(9)^
5. The CREIGHTON MODEL follow-up form (FUF) - standardizes teaching and knowledge transfer between practitioner and users, thus ensuring equal access to vital information for the appropriate use of the system.^(2)^

The user education system begins with an Introductory Session (IS), which can take place in a group or individual setting. This is the moment where the registration form is filled out. After the IS, there are 8 individual Follow-up (FU) between the practitioner and the user, over the course of a year. The first 4 FUs take place every 15 days, the fifth FU after 30 days, and the remaining ones every 3 months. After the eighth, a FU is suggested every 6 or 12 months.

In each of these FU, the practitioner applies a Follow-up Form (FUF) for each user and fills it out in printed form during each FU.^(13)^ The FUF ensures that users have adequate and standardized access to information and instructions for recording their menstrual cycle in a chart.^(2)^ The FUF consists of 25 sections distributed as follows: The face sheet, Completion of forms section, All medications, smoking, use of alcohol section, Comments section, and 21 additional numbered sections.

With each FU, filling out the 25 sections generates various pieces of information. These include demographic data, the user's health history, CrMS learning path, adherence, and intention of use of the method. The intention of use can be to achieve or delay a pregnancy or to monitor gynecological health. For this reason, it is essential that the content used in this form be clear, applicable, and effective in its function,^(2)^ both for the practitioner and the user.

Each section of the FUF indicates in which FU it should be applied. Therefore, some sections are always applied, while others are applied in specific FU, such as the second and sixth, for example. There are also sections that are applied only once, in a single FU. Finally, some sections are only applied under specific health circumstances, that is, in certain clinical conditions of the user, such as section 12.

Therefore, during the 8 FUs, the user will systematically learn how to record observed discharge in the chart using the Picture Dictionary, the Vaginal Discharge Recording System, and the corresponding stickers. The user instruction protocol is extensive, and this educational system contributes to the effectiveness of the CrMS.^(13)^

Since its creation, the FUF has undergone changes to become an improved and effective educational tool, serving both the practitioner and the user of the CrMS.^(2)^ Currently, it is in its fourth version, printed in 2019, in English. In addition to English, this material has been translated and adapted into several other languages, such as Hungarian, Italian, Spanish, Irish, European Portuguese, and French.^(1)^ However, these were basic translations, not relying on solid methodology and without assessing the results of the final material's use. There are no published studies related to its translation or cultural adaptation.

In Brazil, the material is still translated from English by the practitioner during the FU, highlighting the need for a more accurate cultural translation and adaptation to ensure the understanding and standardization of the method across the country.

This study proposes the translation and cultural adaptation into Brazilian Portuguese of the Follow-up Form (FUF) of the Creighton Model FertilityCare^TM^ System (CrMS). The goal is to enhance the comprehension and application of the method within the Brazilian context, ensuring that all users have access to clear and standardized information.

## Methods

This was a methodological study focused on the translation and cultural adaptation of the Follow-up Form (FUF) from the Creighton Model FertilityCare^TM^ System (CrMS) for use in Brazilian Portuguese. The volunteers who agreed to participate in the study signed the Free and Informed Consent Form in a virtual format.

The process of translating and culturally adapting the FUF for use in Brazil was carried out with the author's permission, following the methodology proposed by the World Health Organization.^(15)^ This methodology consists of six steps: Translation, Expert Panel, Back Translation, Pre-testing, Review, and Final Version. Its goal is to achieve a cross-cultural and conceptually equivalent version to the original English version.^(15)^

The first phase was the translation of the original version into Portuguese by a translator familiar with the English language but whose native language is Portuguese. In this step, the focus was on the conceptual rather than literal translation of terms to facilitate understanding by the audience. The version created in this stage was called FUF 1.0.

Immediately after the initial translation, the first review was conducted with a panel of experts. This panel was composed of the translator from the first stage, one physician and two practitioners trained in CrMS and certified by the American Academy of FertilityCare^(16)^ Professionals, with one of them holding a bachelor's degree, a teaching degree, and a master's in applied linguistics. In this phase, the 25 sections of the FUF were read by the panel of experts, and each sentence was reviewed to check for any inappropriate expressions or concepts in the translation, as well as any discrepancies between the direct translation and the existing previous version, resulting in the FUF 1.1 version.

The FUF 1.1 version was submitted to back translation, back into the original language (English), by an independent translator whose native language is English and who had no knowledge of the FUF, producing the version called FUF 1.2. In this version, the discrepancies were discussed by the expert panel, resulting in the appropriate version, FUF 1.3.

This latest questionnaire, FUF 1.3, was administered as a pre-test instrument, at least 20 times in each section, to users of the CrMS. The suggestions made during the pre-test phase were analyzed and allowed for the construction of the final version, FUF 1.4. In all phases, a numbering system was developed to track the different versions of the translated documents and to mark revisions of the changes made by the team.

This study complied with all the criteria established by Resolution No. 466/2012 of the National Health Council and was approved by the Research Ethics Committee of the Federal University of São Paulo (UNIFESP), *Certificado de Apresentação de Apreciação Ética* (CAAE) 65759122.9.0000.5505, opinion N° 6.062.414.

## Results

The initial version of FUF 1.0, after translation and review by the panel of experts, had 11 out of the 25 sections considered equivalent, and 14 sections were discussed to resolve discrepancies. These 14 sections are as follows: The face sheet, Completion of forms section, All medications, smoking, use of alcohol section, and sections 3, 4, 5, 6, 7, 8, 11, 12, 13, 14, and 20. The discrepancies were resolved by consensus, considering the theoretical framework and the Brazilian context, resulting in version FUF 1.1. This version was subjected to back-translation, producing FUF 1.2, which showed high equivalence with the original FUF. The back-translation focused on conceptual and cultural equivalence, making a new translation unnecessary. The contributions and changes throughout the process, from the initial translation to version FUF 1.4, are detailed in chart 1.

**Chart 1:** Follow-up Form sections that were adapted during the translation and cultural adaptation process

Item	Original Version 1.0	Translation Version 1.1	Back Translation 1.2	Pre-test Version 1.3	Final Version 1.4
The Face Sheet	Follow up form	Ficha de acompanhamento	Follow Up Form	Formulário de acompanhamento	Formulário de acompanhamento
The Face Sheet	If married, length of present marriage	Caso sejam casados indique o tempo de casamento atual	If currently married, how long	Se casados, duração do casamento atual	Se casados, duração do casamento atual
The Face Sheet	Number of marriage for each	Quantidade de vezes que cada um se casou	Number of marriages each	Número de casamentos de cada um	Número de casamentos de cada um
The Face Sheet-3	Number of children now living	Número atual de filhos vivos	Current No. of living children	Número de filhos atualmente vivos	Número de filhos atualmente vivos
The Face Sheet-7 1s	Regular Cycles – sterilized	Ciclos regulares - estéril	Regular-sterile cycles	Ciclos regulares-esterilizada	Ciclos regulares-esterilizada
The Face Sheet 7-5	Post-pill (within past year)	Pós pílula (no último ano)	Post-pill (last year)	Pós-pílula (dentro do último ano)	Pós-pílula (dentro do último ano)
The Face Sheet 7-7	Postpartum - not breastfeeding	Pós parto - não está amamentando	Postpartum – not breastfeeding	Pós-parto – não amamentando	Pós-parto – não amamentando
3	Additional persons at follow -up	Outras pessoas no acompanhamento	Additional people in the follow-up	Pessoas adicionais no acompanhamento	Pessoas adicionais no acompanhamento
Completion of forms	Completion of forms	Conclusão das fichas	Conclusion of tracking sheets	Conclusão dos formulários	Conclusão dos formulários
Completion of forms – 1	General intake form	Ficha geral de entrada	General Entry Form	Formulário geral de entrada	Formulário de inscrição
Completion of forms – 2	Program evaluation forms	Fichas de avaliação do programa	Program evaluation sheets	Formulários de avaliação do programa	Formulário de avaliação do programa
Completion of forms – 3	Have you read Introductory Booklet?	Vocês leram a apostila de introdução?	Did you read the Introductory Handout?	Vocês leram a apostila introdutória?	Vocês leram o manual introdutório?
All medications, smoking, use of alcohol	All medications, smoking, use of alcohol	Medicamentos, tabagismo, ingestão de bebida alcoólica	All Medications, smoking, alcoholic beverage consumption	Todos os medicamentos, tabagismo e ingestão de bebida alcoólica	Todos os medicamentos, tabagismo e ingestão de bebida alcoólica
All medications, smoking, use of alcohol	Herbs	Ervas	Herbs	Ervas	Fitoterápico
All medications, smoking, use of alcohol	Question: since your last follow up, are there any new medications, vitamins, or herbs you have taken?	Pergunta: Desde o seu último acompanhamento você tomou algum medicamento, vitamina ou erva?	Question: Since your last follow-up, have you taken any new medication, vitamins, or herbs?	Pergunta: Desde o seu último acompanhamento, há novos medicamentos, vitaminas ou ervas que tenha tomado?	Desde o seu último acompanhamento, há novos medicamentos, vitaminas ou fitoterápico que tenha tomado?
4	Observations - review and assessment	Observações - reavaliação e avaliação	Observations - review and evaluation	Observações revisão e avaliação	Observações revisão e avaliação
4	Reviewed	Revisto	Revised	Revisado	Revisado
4	Reasons for observational routine explained (First fu - when reviewed)	Explicações das razões para rotina observacional (Primeiro fu - quando reavaliado)	Reasons for observational routine explained (First Follow-up – when reviewed)	Razões para a rotina observacional explicadas (Primeiro Acompanhamento– quando revisado)	Razões para a rotina observacional explicada (Primeiro Acompanhamento– quando revisado)
4 A	Do you use flat layers of tissue?	Você usa lenço de papel liso?	Do you use flat sheets of toilet paper?	Você usa camadas lisas de papel higiênico?	Você usa camadas planas de papel higiênico?
4 B	Do you ever use crumple tissue?	Você sempre usa lenço de papel amassado?	Do you, at times, use crumpled toilet paper?	Você eventualmente usa o papel higiênico amassado?	Você eventualmente usa o papel higiênico amassado?
4 D	Do you check from the urethra thru the perineal body?	Você verifica da uretra até a região perineal?	Do you check from the urethra to the perineal region?	Você verifica da uretra até a região perineal?	Você verifica da uretra até a região perineal?
4 E	Do you always wipe until the mucus is gone? Until dry?	Você sempre se limpa até que não haja mais muco? Até estar tudo seco?	Do you always clean until there is no more mucus? Until dry?	Você sempre se limpa até que não haja mais muco? Até ficar seca?	Você sempre se limpa até que não haja mais muco? Até ficar seca?
4 F	Do you ever do internal examinations to check for the mucus?	Você realiza constantemente exames internos para análise do muco?	Do you, at times, have internal exams for mucus examinations?	Você eventualmente realiza exames internos para verificar o muco?	Você eventualmente realiza exames internos para verificar o muco?
4 G	Do you ever check directly with your fingers?	Você analisa constantemente com seus dedos?	Do you, at times, examine directly with your fingers?	Você eventualmente verifica direto com seus dedos?	Você eventualmente verifica direto com seus dedos? Sem usar papel higiênico?
4 H	Do you ever base your observations on what you may see on your underwear?	Você sempre baseia suas observações naquilo que vê em sua calcinha?	Do you, at times, base your observations on what you see in your panties?	Você eventualmente baseia suas observações naquilo que vê em sua calcinha?	Você eventualmente baseia suas observações naquilo que vê em sua calcinha?
4 I	Do you check every time before urination?	Você observa todas as vezes antes de urinar?	Do you examine every time before urinating?	Você verifica todas as vezes antes de urinar?	Você verifica todas as vezes antes de urinar?
4 J	Do you check every time after urination?	Você observa todas as vezes depois de urinar?	Do you examine every time after urinating?	Você verifica todas as vezes depois de urinar?	Você verifica todas as vezes depois de urinar?
4 K	Do you check every time before a bowel movement?	Você observa todas as vezes antes do intestino movimentar?	Do you examine every time before a bowel movement?	Você verifica todas as vezes antes de evacuar?	Você verifica todas as vezes antes de evacuar?
4 L	Do you check every time after a bowel movement?	Você observa todas as vezes depois do intestino movimentar?	Do you examine every time after a bowel movement?	Você verifica todas as vezes depois de evacuar?	Você verifica todas as vezes depois de evacuar?
4 M	Is there ever a time when you go to the bathroom but do not check?	Existem vezes em que você vai ao banheiro mas não observa?	Do you ever go to the bathroom and not examine?	Alguma vez você vai ao banheiro, mas não verifica?	Alguma vez você vai ao banheiro, mas não verifica? Nem antes, nem depois?
4 N	Do you check every time before going to bed?	Você observa todas as vezes antes de ir para cama?	Do you examine every time before going to sleep?	Você verifica todas as vezes antes de ir dormir?	Você verifica todas as vezes antes de ir dormir?
4 O	Do you bear down every time before going to bed? (within 15 min)	Você sempre faz força para baixo antes de ir para cama? (15 min antes)	Do you try to use force (similar to a bowel movement) every time before going to sleep? (up to 15 min before)	Você realiza força semelhante à evacuação todas as vezes antes de ir dormir? (até 15min antes)	Você realiza força semelhante à evacuação todas as vezes antes de ir dormir? (até 15min antes)
4 P	Do you make a decision at each observation?	Você toma alguma decisão a cada observação?	Do you make any decisions with each observation?	Você toma uma decisão a cada observação?	Você toma uma decisão a cada observação?
4 Q	Do you register that observation?	Você registra a observação?	Do you record this observation?	Você registra essa observação?	Você registra essa observação?
4 R	Do you ever discontinue your observation?	Você interrompe suas observações algumas vezes?	Do you, at times, interrupt your observations?	Você eventualmente interrompe suas observações?	Você, eventualmente, deixa de se observar?
4 S	Do you ever get complacent about your observations?	Você sempre se queixa sobre as suas observações?	Do you, at times, get displeased with your observations?	Você eventualmente fica displicente quanto às suas observações?	Você eventualmente fica desatenta quanto às suas observações?
4	The Three Steps	As três etapas	The three steps	Os três passos	Os três passos
4 T	Can you tell me what are the three steps in checking for the mucus ?	Você consegue me dizer quais são as três etapas para verificação do muco?	Can you tell me the three steps for mucus examination?	Você pode me dizer quais são os três passos para a verificação do muco?	Você pode me dizer quais são os três passos para a verificação do muco?
4 V	Do you always finger test anything you see on the tissue?	Você sempre faz o teste do dedo antes de observar o papel higiênico?	Do you always perform the finger test before looking at the toilet paper?	Você sempre faz o teste do dedo antes de observar o papel higiênico?	Você sempre faz o teste do dedo em qualquer coisa que você veja no papel higiênico?
4 X4	How often do you go swimming? (#of time) Do you check before and after?	Com que frequência você entra na piscina? (Número de vezes) Você verifica antes e depois?	How often do you do you get in the pool ?	Com que frequência você nada? (n° de vezes) Você verifica antes e depois?	Com que frequência você nada? (n° de vezes) Você verifica antes e depois?
5A	Vagina is a self cleansing organ	Vagina é um órgão que se limpa sozinho	Is the vagina a self-cleaning organ?	A vagina é um órgão que se auto higieniza	A vagina é um órgão que se auto higieniza
5 B	There is no need to douche	Não há necessidade de lavar com ducha	There is no need for douching	Não há necessidade de ducha higiênica	Não há necessidade de ducha higiênica
5 C	Do you use tampons (1), pads (2), or minipads (3)?	Você usa absorventes internos (1), absorventes higiênicos (2) ou mini absorventes (3)?	Do you use tampons (1), sanitary napkins (2) or mini tampons (3)?	Você usa absorventes internos (1), absorventes higiênicos (2) ou mini absorventes (3)?	Você usa absorventes internos (1), absorventes externos (2) ou protetor diário (3)?
5 D	Do you use scented tampons (1), pads (2), or minipads (3)?	Você usa absorventes internos, absorventes higiênicos ou mini absorventes perfumados?	Do you use any tampons, hygienic tampons, or mini tampons that are scented?	Você usa absorventes internos, absorventes higiênicos ou mini absorventes perfumados?	Você usa absorventes internos (1), absorventes externos (2) ou protetor diário (3) perfumados?
5 F	Do you use scented or dyed toilet tissue?	Você usa papel higiênico perfumado ou colorido?	Do you use scented or colored toilet paper?	Você usa papel higiênico perfumado ou colorido?	Você usa papel higiênico perfumado ou colorido?
5 G	Do you use fabric softeners in the dryer?	Você usa amaciante de roupas na máquina de lavar?	Do you use fabric softener in the dryer?	Você usa amaciante de roupas na secadora?	Você usa amaciante de roupas na secadora?
5 J	Do you use cotton-crotch panty hose?	Você usa meia-calça de algodão?	Do you wear cotton pantyhose?	Você usa meia-calça de algodão?	Você usa meia-calça de algodão?
6	Picture dictionary presented	Dicionário ilustrado apresentado	Image dictionary presented	Dicionário de imagens apresentado	Dicionário de imagens apresentado
7 D	What is the pre-Peak phase of the cycle?	O que é fase pré-ovulatória ou folicular do ciclo?	What is the pre-Peak phase of the cycle?	O que é a fase pré-ápice do ciclo?	O que é a fase pré-ápice do ciclo?
7 E	What is the post-Peak phase of the cycle?	O que é fase pós-ovulatória ou lútea do ciclo?	What is the post-Peak phase of the cycle?	O que é a fase pós-ápice do ciclo?	O que é a fase pós-ápice do ciclo?
8 M	Are barrier methods being used?	Métodos contraceptivos estão sendo utilizados?	Are barrier methods being used?	Métodos de barreira estão sendo utilizados?	Métodos de barreira estão sendo utilizados?
8 O	Discuss concomitant use of barrier methods, coitus interruptus and withdrawal	Discussão do uso em conjunto de métodos contraceptivos, coito interrompido e retirada	Discuss joint use of barrier methods and interrupted coitus	Discuta o uso em conjunto de métodos de barreira e coito interrompido	Discuta o uso em conjunto de métodos de barreira e coito interrompido
11 A	Early Ovulation (occurs in short cycles) (PD) Picture Dictionary How would you know you are having an early ovulation?	Ovulação precoce (ocorre em ciclos curtos) (DI) Dicionário ilustrado Como você saberia que está tendo ovulação precoce?	Early ovulation (occurs in short cycles) (ID) Image Dictionary How would you know you are having early ovulation?	Ovulação precoce (ocorre em ciclos curtos) (DI) Dicionário de imagem Como você saberia que está tendo ovulação precoce?	Ovulação antecipada (ocorre em ciclos curtos) (DI) Dicionário de imagem Como você saberia que está tendo ovulação antecipada?
11 A	How would you manage an early ovulation	Como você lida com a ovulação precoce?	How would you deal with early ovulation?	Como você lidaria com a ovulação precoce?	Como você lidaria com a ovulação antecipada?
11 A-3	Observe for mucus/dryness on L, VL, or B days of flow	Observe o muco/secura nos dias de fluxo L, VL ou B	Observe mucus/dryness on flow days L, VL or B	Observe o muco/secura nos dias de fluxo L, VL ou B	Observe presença ou ausência de muco nos dias de fluxo L, VL ou B
11 B	"Double"Peak (PD) What is a "double Peak" and when does it occur?	Dia fértil em "dobro" (DI) O que é o Dia fértil "em dobro" e quando ele ocorre?	"Double"Peak (ID) What is "double Peak" and when does it occur?	"Duplo Ápice" (DI) O que é o "duplo Ápice" e quando ele ocorre?	"Duplo Ápice" (DI) O que é o "duplo Ápice" e quando ele ocorre?
11 B-4	The "second" Peak generally occurs after stress is relieved	O "segundo" dia fértil, em geral, ocorre depois que o estresse passou	The "second" Peak usually occurs after stress is relieved	O "segundo" Ápice, geralmente, ocorre depois que o estresse for aliviado	O "segundo" Ápice, geralmente, ocorre depois que o estresse for aliviado
11 B-5	Ovulation occurs at the "Second" Peak	A ovulação ocorre no "segundo" dia fértil	Ovulation occurs at the "Second" Peak	A ovulação ocorre com o "segundo" Ápice	A ovulação ocorre com o "segundo" Ápice
11 B	When and how would you anticipate a "double" Peak?	Quando e como você prevê um Dia fértil "em dobro"?	When and how would you predict a "double" Peak?	Quando e como você preveria um "duplo" Ápice?	Quando e como você preveria um "duplo" Ápice?
11 B-9	When peak buildup or Peak Day appears unusual	Quando o aumento da fertilidade ou o dia fertil parece incomum	When mucus progression or Peak day seems atypical	Quando a progressão de muco ou o Dia Ápice parecerem atípicos	Quando a progressão de muco ou o Dia Ápice parecerem atípicos
11 B-10	When you are 16 days or more post-Peak (missed period)	Quando você está a 16 dias ou mais no período pós-ovulatório (período perdido)	When you are at 16 days or more in post-Peak (No Menstruation Occurrence)	Quando você está a 16 dias ou mais no pós- Ápice (não ocorrência da menstruação)	Quando você está a 16 dias ou mais no pós- Ápice (não ocorrência da menstruação)
11 B	How would you manage a "Double" Peak?	Como você lida com o Dia fértil "em dobro"?	How would you handle a "double" Peak?	Como você lidaria com o "duplo" Ápice?	Como você lidaria com o "duplo" Ápice?
11 B-11	If "double" Peak anticipated, follow pre-Peak, end-of-day instructions until the situation is clarified	Se o Dia fértil "em dobro" antecipou, siga as instruções do Pré-dia fértil, fim do dia até a situação ser esclarecida	If a "double" Peak is predicted, follow the Pre-Peak end-of-day instructions until the situation clears up	Se o "duplo" Ápice for previsto, siga a instrução fim do dia do Pré-Ápice, até que a situação seja esclarecida	Se o "duplo" Ápice for previsto, siga a instrução fim do dia do Pré-Ápice, até que a situação seja esclarecida
11 B-12	Wife monitors peak buildup or if Peak day is unusual	A mulher monitora o aumento do dia fértil ou se o dia fértil não está usual	The wife monitors mucus progression or if the Peak day is atypical	A esposa monitora a progressão de muco ou se o dia Ápice está atípico	A esposa monitora a progressão de muco ou se o dia Ápice está atípico
11 B-13	Husband monitors stress awareness	O homem monitora a conscientização do estresse	The husband monitors stress awareness	O marido monitora a conscientização do estresse	O marido monitora a conscientização do estresse
11 B-14	Ask yourself and your spouse the "Double" Peak questions on P+3	Faça a você mesma e ao seu cônjuge as perguntas de P+3	Ask yourself and your spouse the "double" Peak Question on P + 3	Faça a você mesma e ao seu cônjuge as perguntas de "duplo" Ápice no P + 3	Faça a você mesma e ao seu cônjuge os questionamentos do "duplo" Ápice no P + 3
11 B-15	Are you asking the "double" Peak question	Você está fazendo as perguntas da Pergunta do dia fértil "em dobro"?	Are you asking the "double" Peak question	Você está fazendo as perguntas da Pergunta Do "duplo" Ápice?	Você está fazendo os questionamentos do "duplo" Ápice ?
12 A-6	May be temporarily anovulatory	Pode ficar temporariamente anovulatória	May become temporarily anovulatory	Pode ficar temporariamente anovulatória	Pode ficar temporariamente anovulatória
12 G-4	Pre-Peak Phase – may be shorter/watch for early ovulation and mucus during menstrual flow	Fase pré-ovulatória – pode ser mais curta/observar ovulação precoce e muco durante o fluxo menstrual	Pre-Peak Phase – may be shorter/watch out for early ovulation and mucus during menstrual flow	Fase pré-Ápice – pode ser mais curta/atente-se para ovulação precoce e muco durante o fluxo menstrual	Fase pré-Ápice – pode ser mais curta/atente-se para ovulação antecipada e muco durante o fluxo menstrual
13 I-5	Intercourse on days of fertility abandons the system as a means of avoiding pregnancy and adopts system as a means of achieving pregnancy	A relação sexual nos dias férteis renuncia o método como meio de evitar a gravidez e o adota como meio de engravidar	Sexual intercourse on fertility days discards the method as a means of postponing pregnancy and adopts it as a means to become pregnant	A relação sexual nos dias de fertilidade renuncia o método como meio de adiar a gravidez e o adota como meio de alcançar a gravidez	A relação sexual nos dias de fertilidade abandona o método como meio de adiar a gravidez e o adota como meio de alcançar a gravidez
14 I	List	Registro	List	Lista	Lista
14 I-B	Chart at the end of your day, every day, and record the most fertile sign of the day	Mapeie diariamente, no fim de cada dia, e registre o sinal mais fértil do dia	Map at the end of your day, every day, and record the most fertile sign of the day.	Mapeie ao final do seu dia, todos os dias, e registre o sinal mais fértil do dia.	Todos os dias, ao final do dia, registre na planilha o sinal mais fértil observado ao longo do dia
14 I-D	Days of fertility (Select to achieve pregnancy)	Dias ferteis (escolha para engravidar)	Fertility days (Select them to become pregnant)	Dias de fertilidade (selecione-os para alcançar a gravidez)	Dias de fertilidade (selecione-os para alcançar a gravidez)
14 I D-1	The menstrual flow	O fluxo menstrual	The menstrual flow	O fluxo menstrual	Período de fluxo menstrual
14 I D-2	From beginning of mucus until 3 full days past the peak	Do início do muco até 3 dias inteiros após o dia fértil	From mucus onset to three full days after the Peak	Desde o início do muco até três dias completos após o ápice	Assim que aparece o muco até 3 dias completos após o ápice
14 I D-3	1 or 2 days of Non-Peak mucus Pre-Peak	O muco de 1 ou 2 dias não férteis na fase pré-ovulatória	1 or 2 days of Non-Peak mucus in Pre-Peak phase	1 ou 2 dias de muco não-ápice na fase pré-ápice	1 ou 2 dias de muco tipo não-ápice na fase pré-ápice
14 I D-4	Or 3 more days of Non-Peak mucus Pre-Peak – plus count 3	O muco de 3 ou mais dias não férteis na fase pré-ovulatória – somando 3	Or 3 more days of Non-Peak mucus in the Pre-Peak phase – count 3 more	3 ou mais dias de muco não-ápice na fase pré-ápice – conte mais 3	3 ou mais dias consecutivos de muco tipo não-ápice na fase pré-ápice – conte mais 3
14 I-D 5	Any single day of Peak mucus – plus count 3	O muco de qualquer dia fértil – somando 3	Any day other than Peak mucus – count 3 more	Qualquer dia isolado de muco ápice – conte mais 3	Qualquer dia isolado de muco tipo ápice – conte mais 3
14 I D-6	Any unusual bleeding – plus count 3	Qualquer sangramento incomum – somando 3	Any abnormal bleeding – count 3 more	Qualquer sangramento não usual – conte mais 3	Qualquer sangramento não usual – conte mais 3
14 F	Seminal Fluid Instruction (see manual)	Orientação sobre o semem (veja o manual)	Seminal Fluid Collection Instruction (see manual)	Instrução do fluido seminal (veja o manual)	Instrução do fluido seminal (veja o manual)
14 G	"Double" Peak	Dia fertil em "Dobro"	"Double" Peak	"Duplo" Ápice	"Duplo" Ápice
14 G-2	If the Post-Peak phase greater than 16 days in duration and system used properly to avoid pregnancy, anticipate "missed"period from of "double" Peak	Se a fase pós-ovulatória tiver duração maior que 16 dias e o método for utilizado corretamente para evitar a gravidez antecipe o uso do período "perdido" do dia fértil "em dobro"	If the Post-Peak phase lasts longer than 16 days and the system has been used correctly to delay pregnancy, assume a "double" Peak due to no menstruation	Se a fase pós-ápice tiver duração superior a 16 dias e o sistema foi utilizado corretamente para adiar a gravidez, presuma um "duplo" ápice devido a não ocorrência da menstruação	Se a fase pós-ápice for superior a 16 dias e o método foi usado corretamente para adiar a gravidez, presuma um "duplo" ápice devido a não ocorrência da menstruação
14 G-3	When anticipating "Double" Peak, keep to the end of the infertile days and continue with good observations.	Quando antecipar o dia fértil "em dobro", mantenha o registro até o término dos dias não férteis e continue as boas observações.	Assuming a "Double" Peak, follow the instruction at the end of the infertile days and continue with good observations.	Ao presumir um "Duplo" Ápice, mantenha a instrução do final dos dias inférteis e continue com boas observações	Ao presumir um "Duplo" Ápice, mantenha a instrução do final dos dias inférteis e continue com boas observações
14 H	When in doubt, consider yourself of Peak fertility and count 3	Se estiver em dúvida, considere-se no dia fértil e some 3	When in doubt, consider yourself with Peak fertility and count 3	Quando estiver em dúvida, considere-se com fertilidade do tipo ápice e conte 3	Quando estiver em dúvida, considere-se com fertilidade do tipo ápice e conte 3 dias de fertilidade na sequência
14 I-2	Use days of greatest quantity and quality and first two days afterward.	Utilize dias de maior quantidade e qualidade de muco e os dois dias subsequentes	Use higher-quality mucus days, and quality of mucus and both days subsequent.	Utilize dias de maior quantidade e qualidade de muco e os dois dias subsequentes	Selecione dias de maior quantidade e qualidade de muco e os dois dias subsequentes
14 I-3	Record the amount of stretch of the mucus (1", 2", 3" etc)	Registre o tamanho da elasticidade do muco (2,5cm;5cm;7,5cm etc.)	Record the size of mucus elasticity (2.5cm; 5cm; 7.5cm etc).	Registre o tamanho da elasticidade do muco (2,5cm;5cm;7,5cm etc.)	Registre o tamanho da elasticidade do muco (2,5cm;5cm;7,5cm etc.)
14 I-4	Record abdominal pain (AP), right abdominal pain (RAP), and left abdominal pain (LAP)	Registre dor abdominal (AP), dor abdominal à direita (RAP), e dor abdominal à esquerda (LAP)	Record abdominal pain (AP), right abdominal pain (RAP), and left abdominal pain (LAP)	Registre dor abdominal (AP), dor abdominal à direita (RAP), e dor abdominal à esquerda (LAP)	Registre dor abdominal (AP), dor abdominal do lado direito (RAP), e dor abdominal do lado esquerdo (LAP)
14 J	Essential sameness question – "Is today essentially the same as yesterday?"	As mesmas perguntas essenciais "Hoje é essencialmente igual a ontem?"	Question the essential similarity – "Is today essentially similar to yesterday?"	Pergunta sobre a semelhança essencial – "Hoje é essencialmente semelhante a ontem?"	Pergunta da semelhança – "Hoje é essencialmente igual a ontem?"
14 K	Yellow Stamp Instructions	Instruções com marcação amarela	Yellow Adhesive Instructions	Instruções sobre adesivo amarelo	Instruções dos adesivos amarelos
14 K-5	Discontinue use when period starts (BF)	Descontinue o uso quando a menstruação começar (amamentação)	Discontinue use when menstruation begins (BREASTFEEDING)	Descontinue o uso quando a menstruação começar (amamentação)	Descontinue o uso ao vir a menstruação (amamentação)
14 K-6	Discontinue Pre-Peak Y.S. in regular cycles when mucus cycle < 9 days	Descontinue adesivos amarelos na fase pré-ovulatória em ciclos regulares quando o ciclo do muco for < que 9 dias	Discontinue yellow adhesives in Pre-Peak phase at regular cycles when the mucus cycle is < 9 days	Descontinue os adesivos amarelos na fase pré-ápice em ciclos regulares quando ciclo de muco < 9 dias	Descontinue os adesivos amarelos na fase pré-ápice em ciclos regulares com ciclo de muco < 9 dias
14 M	End of day instructions apply through the first normal menstrual cycle	As instruções para o fim do dia aplicam-se ao primeiro ciclo menstrual normal	End-of-day instructions apply throughout the first normal menstrual cycle	As instruções do fim do dia aplicam-se ao longo do primeiro ciclo menstrual normal	As instruções do final do dia aplicam-se ao longo do primeiro ciclo menstrual normal
14 N	Other (e.g. second stretch test, second wipe test, , etc.)	Outro (p.ex. teste do semen, segundo teste da limpeza, segundo teste da viscosidade, etc.)	Other (e.g., second stretch test, second-pass toilet paper test, etc.)	Outro (p.ex. teste da segunda esticada, teste da segunda passada do papel higiênico, etc.)	Outros (p.ex. teste da segunda esticada, teste da segunda passada do papel higiênico, etc.)
20	Change of Status	Mudança de Condição	Status Change	Mudança de Status	Mudança de Status
20 1	Transferred to other CrMS provider	Transferido para outro agente do CrMS	Transferred to another CrMS agent	Transferido para outro agente do CrMS	Transferido para outro profissional do CrMS

**Figure 1:**
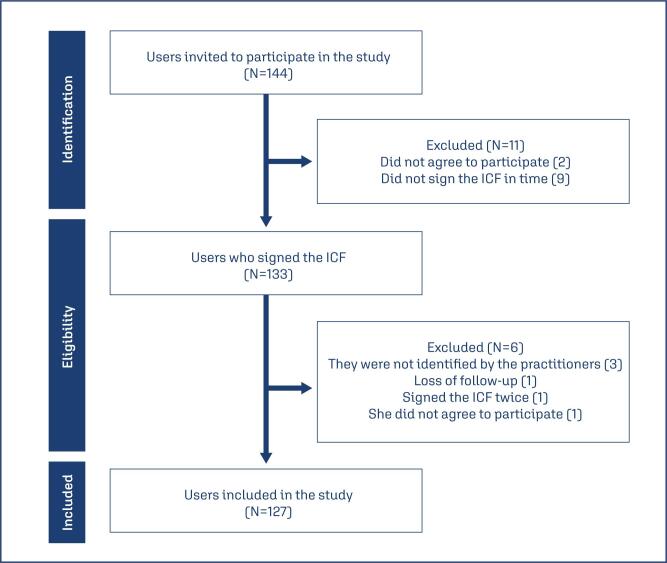
Flowchart of the selection of CrMS users for inclusion in the study

Version FUF 1.3 was applied as a pre-test instrument to the target population. Three Brazilian practitioner trained by CrMS and certified by the American Academy of FertilityCare Professionals, who were not part of the expert panel, applied FUF 1.3 during FU. This was conducted in a private network with users who agreed to participate in the study and signed the informed consent form (ICF). Between July and December 2023, 144 users were approached for the application of FUF 1.3, of whom 127 were included in the analysis (Figure 1).

The average age of the users was 33.7 years, ranging from 20 to 46 years, and 112 (88.2%) were married (Table 1). Among the 127 users served, 126 resided in Brazil and 1 in England. Regarding the distribution across the national territory, 15 different Brazilian states and the Federal District were represented, with 74 (58.3%) of the CrMS users residing in São Paulo. The states represented with the respective number of users were: Espírito Santo 1 (0,8%), Mato Grosso 1 (0,8%), Pernambuco 1 (0,8%), Rondônia 1 (8,0%), Santa Catarina 1 (0,8%), Distrito Federal 2 (1,6%), Mato Grosso do Sul 2 (1,6%), Pará 2 (1,6%), Paraíba 3 (2,4%), Ceará 4 (3,1%), Rio Grande do Sul 4 (3,1%), Paraná 6 (4,7%), Goiás 7 (5,5%), Rio de Janeiro 8 (6,3%), Minas Gerais 9 (7,1%).

**Table 1:** Characterization of the study population

Variables		n(%)
Age group		
	10 to 20 years	1(0,8)
	21 to 30 years	39(30,7)
	31 to 40 years	71(55,9)
	41 to 50 years	15(11,8)
	Missing data	1(0,8)
Marital status		
	Married	112(88,2)
	Engaged	8(6,3)
	Single	7(5,5)
Initial reproductive category		
	Infertility	61(48,0)
	Regular cycles	30(23,6)
	Exclusive lactation	12(9,5)
	Partial lactation	6(4,7)
	Pre-menopause	5 (3,9)
	Long cycles	5(3,9)
	Post-pill	3(2,4)
	Post-abortion	2(1,6)
	Pregnant	0(0)
	Post-partum	0(0)
	Data missing	3(2,4)
Last method of family planning used		
	None	69(54,4)
	CrMS	17(13,4)
	Billings ovulation method	9(7,1)
	Calendar method	9(7,1)
	Withdrawal	5(3,9)
	Natural cycle	2(1,6)
	Abstinence	1(0,8)
	Pill	3(2,3)
	Intrauterine device (IUD)	3(2,3)
	Condom	3(2,3)
	Tubal ligation	1(0,8)
	Vasectomy	1(0,8)
	Chastity	2(1,6)
	Not reported	2(1,6)

As soon as each user starts using the CrMS, she is classified according to her initial reproductive category, which guides the teaching process and the selection of some specific FU subsections of the FUF. This information is relevant because it determines the necessary educational information, such as that in section 12, making the CrMS personalized for each user. The category can change over time and is documented in the FUF.

Table 1 shows the classification of the initial reproductive category of the 127 users. The infertility category includes women who have not become pregnant in the past 12 months’ despite having regular sexual intercourse without using contraceptive methods. The regular cycles category includes women whose predominant cycle pattern ranges from 21 to 38 days. The long cycles category refers to women with a predominant cycle pattern longer than 38 days. The pre-menopause category includes women aged 40 and over who do not wish to become pregnant. The post-pill category consists of women who have chosen to discontinue the use of hormonal methods in the past year. The information reported about the last family planning method used before starting CrMS is described in table 1.

Each CrMS user went through the educational system with an introductory section followed by 8 FU with their practitioner, which occur systematically over the course of a year and then every 6 or 12 months. During the study period, the number of FUs that users had varied as follows: most users had 1 FU, followed by 2 FUs, 5 FUs, 3 FUs, 6 FUs, and 4 FUs, respectively (Table 2). Regarding the sequence of FUs, a large portion of users, 76 (59.84%), were between the first and fifth FUs, 23 (18.11%) were between the sixth and eighth FUs, and 28 (22.05%) users had already surpassed the basic teaching program after the eighth FU, with 2 of them having reached the fourteenth FU.

**Table 2:** Number of users by total follow-up attended during the study period

Number of sessions held	Quantity of users n(%)
1 FU	53(41,7)
2 FU	22(17,3)
3 FU	14(11,0)
4 FU	10(7,9)
5 FU	17(13,4)
6 FU	11(8,6)
TOTAL	127(100)

FU - Follow-up

**Figure 2:**
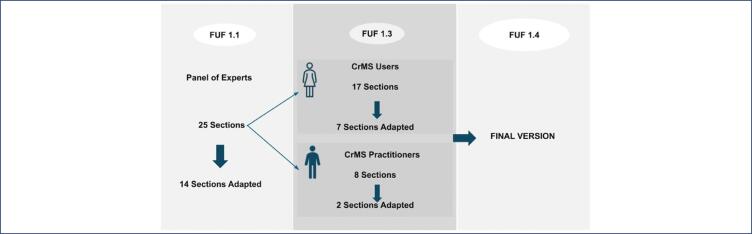
Adaptations made by the panel of experts resulted in version FUF 1.1. Adaptations made by users and practitioners when applied as a pre-test instrument generated version FUF 1.3 which has resulted in the final version FUF 1.4

During the data collection period of the study, it was possible to determine which sections of the FUF were applied in each FU, highlighting the number of times each section of the FUF was applied. Some sections, used in every FU, were applied 330 times. This calculation is possible due to information on the number of users followed and the consideration of which sections of the FUF are applied in each FU. Users who advance further in the process are subjected to the same sections of the form more than once, which is why there is an exponential increase in the number of applications. Among the 25 sections, there are 8 (Completion of forms, 8, 9, 10, 17, 18, 19, and 20) where some items, or the entire section, are never applied or read directly to the users, as they are filled out solely by the practitioner during the FUs. These eight sections were evaluated by the three practitioners during the pre-test phase at each FU. Of these, only two were discussed and adapted (Figure 2), as detailed in chart 1. Thus, out of the 25 sections, 17 are applied directly to the users. The users provided feedback on the overall interpretation of the instrument and on each item. Of these 17 sections, only 7 (Completion of forms, all medication, smoking, use of alcohol section, 4, 5, 8, 11, and 12) required additional explanations or interventions by the practitioner (Figure 2), as per chart 1. The suggestions obtained in the pre-test were analyzed and incorporated into the final version, FUF 1.4. Chart 1 details the changes and contributions throughout the adaptation process.

## Discussion

The research conducted brought significant benefits to the national context, given the impact of CrMS on monitoring fertility and women's gynecological health. The adaptation of educational tools to the local language and culture is crucial for effective learning. This study, pioneering in Brazil and worldwide, stands out for adopting standardized translation and cultural adaptation methodologies, a significant advancement compared to previous translations, which were done in a free manner without established academic translation and back-translation^(15)^ methodologies.

The FUF is the teaching tool for practitioners and the learning tool for users, which ensures a standardized form of learning and application of CrMS. This standardization contributes to the effectiveness and efficiency of CrMS. Therefore, it is crucial for it to be well understood and applied by both practitioners and users.

This work follows the WHO guidelines on the translation and adaptation of instruments. According to these guidelines, the minimum number of times each section of the questionnaire to be translated and adapted must be applied is 10.^(15)^ Meanwhile, the guidelines proposed by Beaton et al.^(17)^ suggest 30 to 40 times. The participation of 127 women in the study allowed for 84% of the sections of the FUF to be applied at least 20 times. Some of these 25 sections were applied up to 330 times. This is an adequate sampling to assess the quality of the translation and cultural adaptation. Some subsections of sections 12, 17, 19, and the entirety of section 21 were applied less than 20 times, and subsections of sections 12 and 19 were applied less than 10 times due to the specificity of clinical conditions. However, all sections were reviewed by the practitioners and evaluated by the expert panel. Subsection J of section 12 is normally not used in Brazil but was included in the study to ensure a complete adaptation of the FUF.

It is important to highlight that the users participated in the study voluntarily and were already familiar with CrMS.

As previously presented, during the first phase of translation into Portuguese, FUF 1.0, and the discussion of this version among the expert panel, adaptations were necessary (Chart 1). Since the translator could not have prior knowledge of the CrMS or the form she was translating^3^, some translations were done literally, while others were interpreted by her, as in Section 11, Item B, Subitem 9: *"progressão de muco ou dia ápice"* was translated as *"aumento da fertilidade ou dia fértil".* A few needed adjustments in verb tense, pronouns, and articles to make sense within the context of teaching and learning the CrMS. Therefore, the expert panel, familiar with the concepts, words, and expressions already used by the CrMS, made the necessary adaptations, resulting in the FUF 1.1 version.

This version was back-translated by another translator, who also had no prior knowledge of the CrMS, resulting in the FUF 1.2 version. Most of the form in this version remained the same or very close to the original English version, confirming that the translation process followed a reliable path concerning the words and concepts used. Only a few sections of this version, FUF 1.2, were discussed again by the expert panel during the construction of the FUF 1.3 version, which was applied to the pre-test population (Chart 1).

The FUF 1.3 version was administered to the pre-test population by three certified practitioners who had not participated in the expert panel. As previously mentioned, there are eight sections in which some items or the entire section are never applied or directly read to the users, as they are completed only by the CrMS practitioner during the FU. Even though they were not directly asked to the users, the practitioners read these sections multiple times,^(9)^ and two of them underwent adaptations (Chart 1).

During the application of FUF 1.3, some sections generated questions. The difficulty in understanding the questions was primarily due to four factors:

The first factor was the format of paired and seemingly repetitive questions, which some sections intentionally include to ensure that the CrMS user is indeed understanding and assimilating the concepts necessary to maintain the observation routine and use the method correctly.

The second factor was the difference in cultural realities, hygiene habits, and consumption practices between the United States and Brazil. We understand that these items in the FUF, even though they are not contextualized within the Brazilian cultural reality, should be maintained due to the universality of the method and the internationalization of its users, who may live outside Brazil and encounter scenarios such as colored toilet paper or dryer sheets, for example.

The third factor contributing to users’ difficulty in comprehension was the use of technical terms, such as medical vocabulary like ‘perineum’ and ‘anovulatory.’ These terms were explained and retained, as once understood, the users no longer had any doubts.

The fourth factor was related to sentences that present many concepts related to CrMS use and, therefore, need to be spoken and explained slowly, so that users can review, understand the concept being discussed, and comprehend it correctly.

The necessary adaptations were made after discussions between the expert panel and the practitioners who applied version FUF 1.3 to the pre-test population, resulting in the final version, FUF 1.4.

The slightly higher average age, 33.7 years, and the large percentage of married users, 88.2%, may be related to the fact that 56.7% of users were not using any family planning method when they began learning CrMS. Nearly half of them, 49.2%, had been trying to conceive in the past year.^(18,19)^

Although 15 Brazilian states and the Federal District were represented by the users, 58.3% of them resided in the State of São Paulo. The three practitioners who applied FUF version 1.3 to the pre-test population also reside in São Paulo. Despite the FUs being online and standardized across the country, the practitioner's location may have influenced the users’ choice.

The teaching system includes 8 FUs over the course of one year, following a standardized sequence of intervals between them, and then every 6 months or annually thereafter. Since the FUF was administered to the pre-test population over a period of 6 months, the maximum number of Follow-up appointments possible for the same user during this study period was 6, provided that the user started the CrMS as soon as she joined the study and followed the recommended intervals between appointments. Therefore, a small number of participants in the study, 38 (29.9%), had between 4 to 6 FUs during this period. However, it is noteworthy that during this study period, it was possible to include both users in the initial learning phase—76 (59.84%) were between the first and fifth FU—and those in the advanced phase—28 (22.05%) users who had already completed the basic teaching program of the CrMS after the eighth appointment.

The standardized way in which the FUF is applied, with the practitioner asking questions directly to the user and her responding immediately, makes it clear whether the question was understood and what difficulties were encountered. Each section is applied to the same client at least twice, most often four times, with intervals between applications varying from 15, 30, 90, to 180 days. This means that users go through the same section multiple times within a short period at the beginning of their learning and later over a longer period, allowing us to suggest a high level of reliability in the translated and applied version of the FUF.

The Brazilian version of the FUF was developed and applied to CrMS users. Understanding the users’ interpretation of each question and making adjustments was essential to identifying issues and enabling question adjustments, thereby improving comprehension and ensuring that the concepts of the original English version were preserved.

A limitation of the study was the inclusion of new users after the data collection period had ended, which prevented their full participation in the study.

## Conclusion

The Follow-up Form for the Creighton Model FertilityCare^TM^ System was culturally adapted for use in Brazil, maintaining intercultural and conceptual equivalence, functioning similarly to the original English version. Thus, the objective of using the WHO's proposal for the translation and cultural adaptation of forms was achieved and confirmed according to the expert committee's evaluation. Therefore, this form can be used by all practitioners in Brazil, with equivalence to the original English version, ensuring that the standardization in the application of the CrMS is maintained throughout the country. This means that there will be an official translation, eliminating the need for each practitioner to translate their material during each appointment, as is currently the case.
